# Supplementary material for: A Rapid Systematic Review Assessing the Effectiveness of Interventions to Promote Self-Management in Workers with Long-Term Health Conditions and Disabilities
Source: Int J Environ Res Public Health. 2024 Dec 23;21(12):1714. doi: 10.3390/ijerph21121714 (PMC11728185; doi:10.3390/ijerph21121714)
Supplement: Supplementary file 1 [file ijerph-21-01714-s001.zip › ijerph-3369621-supplementary.pdf]

Table S1. Full electronic search strategies for all databases.

MEDLINE (via EBSCO host)
(MH “Employee”) OR TI (“Occupational Health” or “Human Resources” or Employee* or Worker* or Occupation* or “Personnel” or Worker* or Workforce* or Manager* or Employer*) OR AB (“Occupational Health” or “Human Resources” or Employee* or Worker* or Occupation* or “Personnel” or Worker* or Workforce* or Manager* or Employer*)
(MH “Self Management”) OR (MH “Self Care”) OR TI (“Self Management” or “Self-Management” or “Self-Monitoring” or “Self Monitoring” or “Self Care” or “Self-Care” or “Supported Employment” or “Social support” or Assistance Program*) OR AB (“Self Management” or “Self-Management” or “Self-Monitoring” or “Self Monitoring” or “Self Care” or “Self-Care” or “Supported Employment” or “Social support” or Assistance Program*)
(MH “Disabled Persons”) OR (MH “Disabled”) OR TI (“Disability” or “Disabled” or “Handicapped” or “People with Disabilities” or “Persons with disabilities” or “Physically Disabled” or “Physically Handicapped” or “Mentally Disabled” or “Mentally Handicapped”) OR AB (“Disability” or “Disabled” or “Handicapped” or “People with Disabilities” or “Persons with disabilities” or “Physically Disabled” or “Physically Handicapped” or “Mentally Disabled” or “Mentally Handicapped”) OR (MH “Chronic Disease”) OR (MH “Multiple Chronic Conditions”) OR TI (Chronic Condition* or Chronic Illness* or “Chronically Ill” or Long-Term Condition* or Long Term Condition* or Long-Term Disorder* or Long Term Disorder* or Long-Term Illness* or Long Term Illness* or “Multiple Chronic Health Conditions” or Multimorbidity* or “Multiple Chronic Illnesses” or “Multiple Chronic Medical Conditions” or “Health Condition” or Health Condition*) OR AB (Chronic Condition* or Chronic Illness* or “Chronically Ill” or Long-Term Condition* or Long Term Condition* or Long-Term Disorder* or Long Term Disorder* or Long-Term Illness* or Long Term Illness* or “Multiple Chronic Health Conditions” or Multimorbidity* or “Multiple Chronic Illnesses” or “Multiple Chronic Medical Conditions” or “Health Condition” or Health Condition*)
(MH “Clinical Trials”) OR (MH “Non-Randomized Controlled Trials”) OR (MH “Cross-Over Studies”) OR (MH “Controlled Before-After Studies”) or (MH “Cohort Studies”) OR (MH “Case-Control Studies “) OR PT (“Randomized Controlled Trial” or “Controlled Clinical Trial” or “Pragmatic Clinical Trial” or “Clinical Trial” or “Observational Study” or “Multicenter Study”) OR AB (“Randomized” or “Randomised” or “Nonrandomized” or “Nonrandomised” or “Randomly” or “Placebo” or Trial* or “Groups”) OR TI (“Quasi Experimental” or “Placebo” or RCT* or “Cross Over” or “Crossover” or “Before After” or “Before and After” or “CBA Study” or “CBA Studies” or “Cohort” or “Concurrent” or “Incidence” or “Follow Up” or “Followup” or “Follow-up” or “Longitudinal” or “Prospective” or “Retrospective” or “Case Control” or “Case Comparison” or “Case Compare” or “Case Base” or Retrospective) OR AB (“Randomized” or “Randomised” or “Nonrandomized” or “Nonrandomised” or “Randomly” or “Placebo” or Trial* or “Groups”) OR TI (“Quasi Experimental” or “Placebo” or RCT* or “Cross Over” or “Crossover” or “Before After” or “Before and After” or “CBA Study” or “CBA Studies” or “Cohort” or “Concurrent” or “Incidence” or “Follow Up” or “Followup” or “Follow-up” or “Longitudinal” or “Prospective” or “Retrospective” or “Case Control” or “Case Comparison” or “Case Compare” or “Case Base” or Retrospective) NOT TI (“Animals” NOT “Humans and Animals”) OR AB (“Animals” NOT “Humans and Animals”)
1 AND 2 AND 3 AND 4
APA PsycINFO/APA PsycARTICLES (via EBSCO host)
(MA “Employee”) OR TI (“Occupational Health” or “Human Resources” or Employee* or Worker* or Occupation* or “Personnel” or Worker* or Workforce* or Manager* or Employer*) OR AB (“Occupational Health” or “Human Resources” or Employee* or Worker* or Occupation* or “Personnel” or Worker* or Workforce* or Manager* or Employer*)
(MA “Self Management”) OR (MA “Self Care”) OR TI (“Self Management” or “Self-Management” or “Self-Monitoring” or “Self Monitoring” or “Self Care” or “Self-Care” or “Supported Employment” or “Social support” or Assistance Program*) OR AB (“Self Management” or “Self-Management” or

“Self-Monitoring” or “Self Monitoring” or “Self Care” or “Self-Care” or “Supported Employment” or “Social support” or Assistance Program*)

(MA “Disabled Persons”) OR (MA “Disabled”) OR TI (“Disability” or “Disabled” or “Handicapped” or “People with Disabilities” or “Persons with disabilities” or “Physically Disabled” or “Physically Handicapped” or “Mentally Disabled” or “Mentally Handicapped”) OR AB (“Disability” or “Disabled” or “Handicapped” or “People with Disabilities” or “Persons with disabilities” or “Physically Disabled” or “Physically Handicapped” or “Mentally Disabled” or “Mentally Handicapped”) OR (MA “Chronic Disease”) OR (MA “Multiple Chronic Conditions”) OR TI (Chronic Condition* or Chronic Illness* or “Chronically Ill” or Long-Term Condition* or Long Term Condition* or Long-Term Disorder* or Long Term Disorder* or Long-Term Illness* or Long Term Illness* or “Multiple Chronic Health Conditions” or Multimorbidity* or “Multiple Chronic Illnesses” or “Multiple Chronic Medical Conditions” or “Health Condition” or Health Condition*) OR AB (Chronic Condition* or Chronic Illness* or “Chronically Ill” or Long-Term Condition* or Long Term Condition* or Long-Term Disorder* or Long Term Disorder* or Long-Term Illness* or Long Term Illness* or “Multiple Chronic Health Conditions” or Multimorbidity* or “Multiple Chronic Illnesses” or “Multiple Chronic Medical Conditions” or “Health Condition” or Health Condition*)

(MA “Clinical Trials”) OR (MA “Non-Randomized Controlled Trials”) OR (MA “Cross-Over Studies”) OR (MA “Controlled Before-After Studies”) or (MA “Cohort Studies”) OR (MA “Case-Control Studies “) OR PT (“Randomized Controlled Trial” or “Controlled Clinical Trial” or “Pragmatic Clinical Trial” or “Clinical Trial” or “Observational Study” or “Multicenter Study”) OR AB (“Randomized” or “Randomised” or “Nonrandomized” or “Nonrandomised” or “Randomly” or “Placebo” or Trial* or “Groups”) OR TI (“Quasi Experimental” or “Placebo” or RCT* or “Cross Over” or “Crossover” or “Before After” or “Before and After” or “CBA Study” or “CBA Studies” or “Cohort” or “Concurrent” or “Incidence” or “Follow Up” or “Followup” or “Follow-up” or “Longitudinal” or “Prospective” or “Retrospective” or “Case Control” or “Case Comparison” or “Case Compare” or “Case Base” or Retrospective) OR AB (“Randomized” or “Randomised” or “Nonrandomized” or “Nonrandomised” or “Randomly” or “Placebo” or Trial* or “Groups”) OR TI (“Quasi Experimental” or “Placebo” or RCT* or “Cross Over” or “Crossover” or “Before After” or “Before and After” or “CBA Study” or “CBA Studies” or “Cohort” or “Concurrent” or “Incidence” or “Follow Up” or “Followup” or “Follow-up” or “Longitudinal” or “Prospective” or “Retrospective” or “Case Control” or “Case Comparison” or “Case Compare” or “Case Base” or Retrospective) NOT TI (“Animals” NOT “Humans and Animals”) OR AB (“Animals” NOT “Humans and Animals”)

1 AND 2 AND 3 AND 4

ProQuest Dissertation & Theses Global

(MH “Employee”) OR TI (“Occupational Health” or “Human Resources” or Employee* or Worker* or Occupation* or “Personnel” or Worker* or Workforce* or Manager* or Employer*) OR AB (“Occupational Health” or “Human Resources” or Employee* or Worker* or Occupation* or “Personnel” or Worker* or Workforce* or Manager* or Employer*)

AND

(MH “Self Management”) OR (MH “Self Care”) OR TI (“Self Management” or “Self-Management” or “Self-Monitoring” or “Self Monitoring” or “Self Care” or “Self-Care” or “Supported Employment” or “Social support” or Assistance Program*) OR AB (“Self Management” or “Self-Management” or “Self-Monitoring” or “Self Monitoring” or “Self Care” or “Self-Care” or “Supported Employment” or “Social support” or Assistance Program*)

AND

(MH “Disabled Persons”) OR (MH “Disabled”) OR TI (“Disability” or “Disabled” or “Handicapped” or “People with Disabilities” or “Persons with disabilities” or “Physically Disabled” or “Physically Handicapped” or “Mentally Disabled” or “Mentally Handicapped”) OR AB (“Disability” or “Disabled” or “Handicapped” or “People with Disabilities” or “Persons with disabilities” or “Physically Disabled” or “Physically Handicapped” or “Mentally Disabled” or “Mentally Handicapped”) OR (MH “Chronic Disease”) OR (MH “Multiple Chronic Conditions”) OR TI (Chronic Condition* or Chronic Illness* or “Chronically Ill” or Long-Term Condition* or Long Term Condition*

or Long-Term Disorder* or Long Term Disorder* or Long-Term Illness* or Long Term Illness* or “Multiple Chronic Health Conditions” or Multimorbidity* or “Multiple Chronic Illnesses” or “Multiple Chronic Medical Conditions” or “Health Condition” or Health Condition*) OR AB (Chronic Condition* or Chronic Illness* or “Chronically Ill” or Long-Term Condition* or Long Term Condition* or Long-Term Disorder* or Long Term Disorder* or Long-Term Illness* or Long Term Illness* or “Multiple Chronic Health Conditions” or Multimorbidity* or “Multiple Chronic Illnesses” or “Multiple Chronic Medical Conditions” or “Health Condition” or Health Condition*)<br><br>AND<br><br>(MH “Clinical Trials”) OR (MH “Non-Randomized Controlled Trials”) OR (MH “Cross-Over Studies”) OR (MH “Controlled Before-After Studies”) or (MH “Cohort Studies”) OR (MH “Case-Control Studies “) OR PT (“Randomized Controlled Trial” or “Controlled Clinical Trial” or “Pragmatic Clinical Trial” or “Clinical Trial” or “Observational Study” or “Multicenter Study”) OR AB (“Randomized” or “Randomised” or “Nonrandomized” or “Nonrandomised” or “Randomly” or “Placebo” or Trial* or “Groups”) OR TI (“Quasi Experimental” or “Placebo” or RCT* or “Cross Over” or “Crossover” or “Before After” or “Before and After” or “CBA Study” or “CBA Studies” or “Cohort” or “Concurrent” or “Incidence” or “Follow Up” or “Followup” or “Follow-up” or “Longitudinal” or “Prospective” or “Retrospective” or “Case Control” or “Case Comparison” or “Case Compare” or “Case Base” or Retrospective) OR AB (“Randomized” or “Randomised” or “Nonrandomized” or “Nonrandomised” or “Randomly” or “Placebo” or Trial* or “Groups”) OR TI (“Quasi Experimental” or “Placebo” or RCT* or “Cross Over” or “Crossover” or “Before After” or “Before and After” or “CBA Study” or “CBA Studies” or “Cohort” or “Concurrent” or “Incidence” or “Follow Up” or “Followup” or “Follow-up” or “Longitudinal” or “Prospective” or “Retrospective” or “Case Control” or “Case Comparison” or “Case Compare” or “Case Base” or Retrospective) NOT TI (“Animals” NOT “Humans and Animals”) OR AB (“Animals” NOT “Humans and Animals”)
Social Care Online (via https://www.scie-socialcareonline.org.uk/search/)
Advanced Search<br><br>All fields “Self-management” AND “worker”.

Table S2. PICOS characteristics of all five studies included in the review.

Authors, year, country	Study design, methods	Population	Intervention(s) and comparator(s)	Outcome measures	Key finding(s)
Haynes et al [44]<br>USA	Clustered RCT<br>Six- and 12-month follow-ups	N = 406 (337 F; 69 M)<br>Mean age = 46 years (SD= 10.8)<br>Ethnicity: White (n= 74); Non-White (n=332)<br>A range of conditions including type i and ii diabetes, asthma, chronic bronchitis, emphysema, or chronic obstructive pulmonary disease, musculoskeletal injury/disorder, digestive diseases/conditions, heart disease, arthritis or other rheumatic disease, cancer, depression, anxiety or other emotional/mental health condition	1. Live Healthy, Work Healthy (LHWH) program* - a version of the CDSMP translated to the workplace<br>2. Delayed control<br><br>*One group-based session (2.5 hours) per week for 6-weeks.	***Work engagement***<br>• Organisational citizenship behaviors<br>• Employee deviant behaviors<br>***Work ability***<br>• Work Ability Index<br>***Job satisfaction***<br>• Turnover intention<br>• Organisational support<br>• Affective organizational commitment<br>• Normative organizational commitment<br>***Psychological wellbeing***<br>• Job stress<br>• Burnout<br>***Work-based attendance***<br>• Absenteeism	The LHWH program had moderate to substantial effects on burnout, work engagement, work ability, affective organizational commitment, organizational citizenship behaviors, and perceived organizational support.
Schopp et al [47]<br>USA	RCT<br>12- and 18-week follow-ups	N = 91 (85 F; 6 M)<br>Mean Age = 46 years (SD= 10.7)<br>Ethnicity: Non-Hispanic White (n= 81); African American (n= 5); Latino (n= 4); American Indian or Alaska Native (n= 1)<br>Chronic conditions not specified	1. Act Healthy (adapted CDSMP* for general employee population) (n= 50)<br>2. Standard care (n= 41)<br><br>*One group-based session (2.5 hours) per week for 6-weeks.	***Self-management***<br>• Self-rated abilities for health practices (SRA) (Confidence in one's ability to perform tasks relating to health)<br>• Health Promoting Lifestyle Profile II (HPLP-II) (frequency of participating in health behaviors, e.g. stress management)	Intervention group had statistically significant increases in the SRA and HPLP-II.
Shaw et al [45]<br>USA	RCT<br>Six-month follow-up	N = 119 (98 F; 20 M)<br>Mean age = 46 years (SD= 12.7)	1. Manage at Work* (n= 60)<br>2. Wait-list control (n= 59)<br><br>*Ten 1-hour, seven 1.5-hour, or five 2-hour group-based	***Work engagement***<br>• The Utrecht Work Engagement Scale (UWES)<br>***Work ability***	The intervention group had an improvement in work-place engagement and better turnover intention. Also showed improvements for self-efficacy, work

		Ethnicity: Asian (n= 2); Black (n= 7); White (n= 108); Not reported (n= 2)<br>A range of chronic conditions including back or neck problems, hand/arm problems leg or feet problems, migraine or severe headaches, vision problems, stomach or bowel disorders, asthma, bronchitis, or emphysema, mental ill-health, hearing problems, cardiovascular disease, severe skin disorders, diabetes)	sessions (specified duration dependent on the schedules/availability of participants).	• Work Limitations Questionnaire (WLQ)<br>***Job satisfaction***<br>• Self-reported job satisfaction<br>• Turnover intention<br>***Workplace fatigue***<br>• Occupational Fatigue Exhaustion Recovery (OFER)<br>***Work self-efficacy***<br>• Combined Pain Self-efficacy questionnaire and Return-to - work self-efficacy scale	fatigue and job satisfaction, but not statistically significant.
Smith et al [46]<br>USA	RCT<br>Six-month follow-up	N = 181 (155 F; 23 M)<br>Mean age = 48 years (SD= 10.1)<br>Ethnicity: Non-Hispanic White (n= 107); Racial/Ethnic Minority (n= 65)<br>A range of chronic conditions including anxiety or other emotional/mental health condition, diabetes, musculoskeletal injury/disorder, depression, arthritis, rheumatic disease, digestive diseases/ conditions, asthma, cancer, heart disease, other physical injuries,	1. CDSMP* (n= 109)<br>2. wCDSMP* (workplace tailored version of CDSMP) (n= 72)<br><br>*One group-based session (2.5 hours) per week for 6-weeks.	***Work engagement***<br>• Work-related stress<br>***Work ability***<br>• Modified Work Ability Index<br>• Work Limitations Questionnaire (short-form)<br>***Work-based attendance***<br>• CDC Healthy Days Scale (2-items)<br>***Condition-specific health-status***<br>• Self-reported levels of stress, pain, fatigue, and sleep problems.<br>• Patient Health Questionnaire-8<br>• Self-Efficacy for managing chronic disease<br>• Medication adherence	The wCDSMP was shown to improve health and work performance among individuals with one or more chronic conditions. Also reduced unhealthy physical days, and fatigue.

		chronic bronchitis,<br>emphysema,<br>or other or chronic obstructive pulmonary disease)			
Wilson et al [48]<br>USA	RCT<br>Six- and 12-month follow-up	N = 411 (341 F; 70 M)<br>Mean age = 46 years (SD= 10.7)<br>Ethnicity: White (n= 255); Black/African American (n= 148); Hispanic (n= 13); Other (n= 8)<br>Chronic conditions not specified	1. LHWH* (n= 130)<br>2. CDSMP* (n= 111)<br>3. Delayed control (n= 170)<br><br>*One group-based session (2.5 hours) per week for 6-weeks.	***Work-based attendance***<br>• CDC Healthy Days Scale (2-items)<br>***Condition-specific health-status***<br>• Medication non-adherence<br>• Self-reported levels of stress, pain, fatigue<br>• Self-Efficacy for managing chronic disease<br>• Health interference	LHWH and CDSMP had positive impacts of health behaviors and self-management. Significant improvements across fatigue, pain, medication adherence for LHWH.<br><br>LHWH is an effective alternative way of delivering the CDSMP.

Table S3. Summary of results for each outcome domain across included studies.

Study	Outcome										
	Primary				***Secondary***						
	Work productivity	Work engagement	Self-management	Workability	Quality of life	Psychological wellbeing	Workplace fatigue	Job Satisfaction	Work-based attendance	Work self-efficacy	Condition-specific health
Haynes et al [44]		↑		↑		↑		↑	--		
Schopp et al [47]			↑								
Shaw et al [45]		↑		--			--	--		--	
Smith et al [46]		↑		--							-↑
Wilson et al [48]									↑		-↑

↑ (*statistically significant positive intervention effect*); -↑ (*mixed effects*); -- (*null effect*); if shaded grey, outcome was not measured.
